# Supplementary material for: Selenium single-wavelength anomalous diffraction de novo phasing using an X-ray-free electron laser
Source: Nat Commun. 2016 Nov 4;7:13388. doi: 10.1038/ncomms13388 (PMC5097167; doi:10.1038/ncomms13388)
Supplement: Supplementary Information — Supplementary Figures 1-4, Supplementary Tables 1-2, Supplementary Note 1 and Supplementary References. [file ncomms13388-s1.pdf]

Supplementary Information

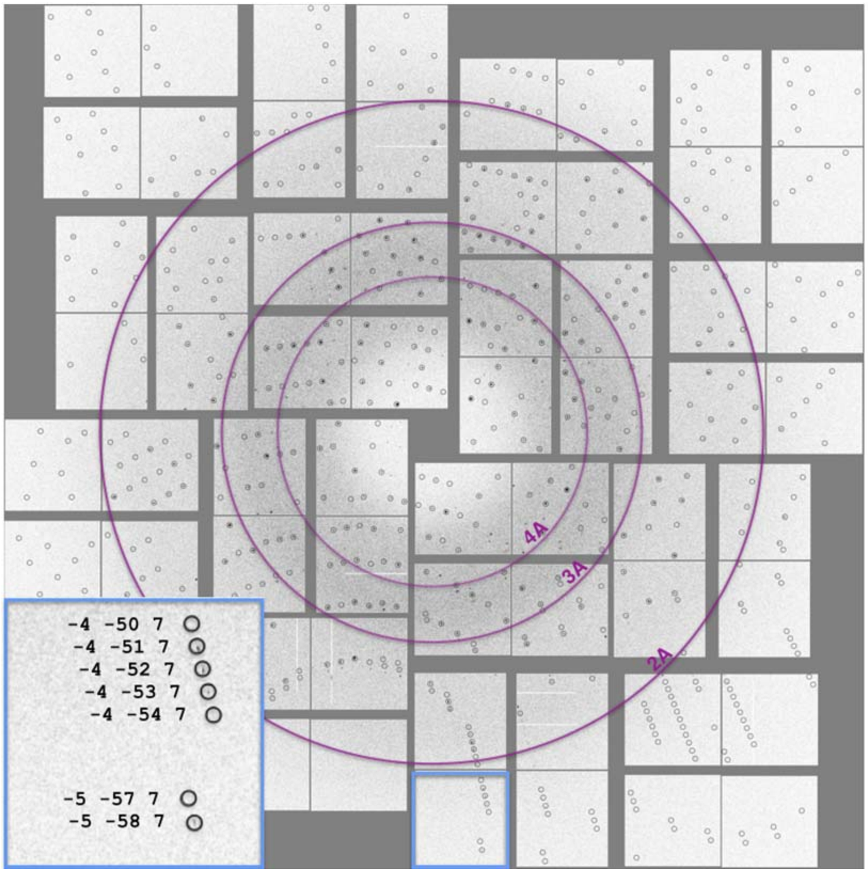

Supplementary Figure 1 | Diffraction pattern with predicted Bragg peak positions (inset) Indexed subpanel.

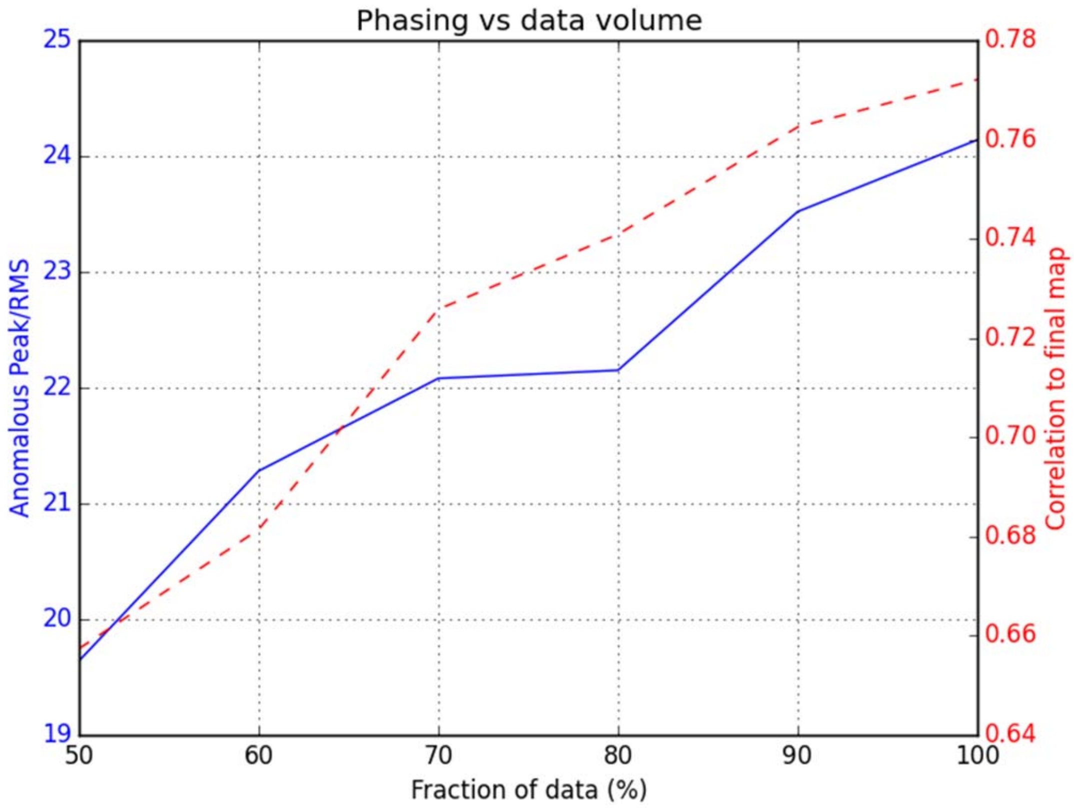

Supplementary Figure 2: Phasing vs data volume. The correlation to final map (red) and anomalous peak RMS (blue) shown as a function of data volume used for the Se-SAD data. Using 100% of the data, only ~0.1% of the 200,000 attempts to phase the data were successful. A key to successful phasing was finding the NCS operator, which only occurred when including the entire data set.

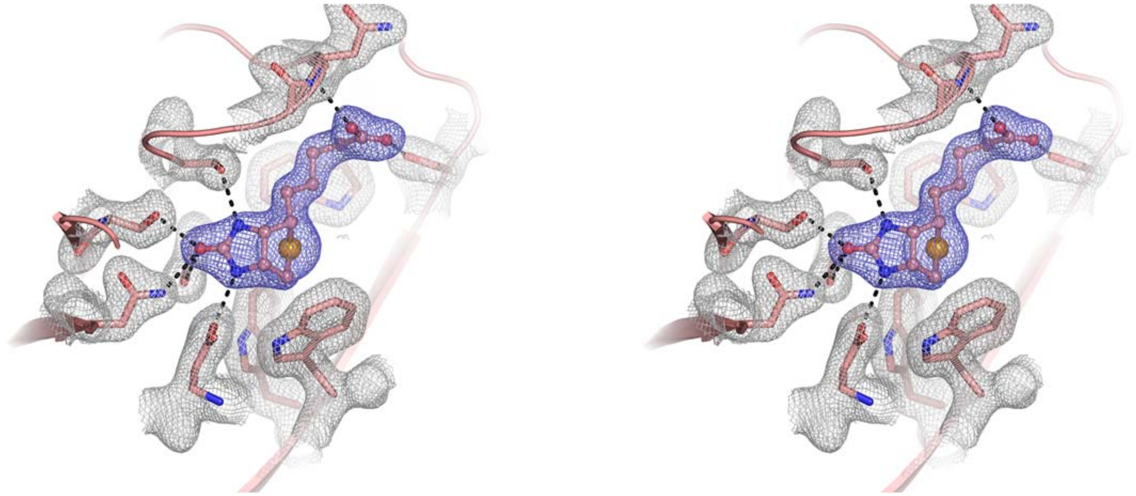

Supplementary Figure 3 | Stereo image of the electron-density map of the selenobiotin binding site. Final $2F_O$-F_C electron-density map of selenobiotin binding pocket contoured at the 1σ level. Selenobiotin is shown in balls and sticks model and its electron-density colored in blue. The locations of active site residues are shown and their electron-density is colored in gray.

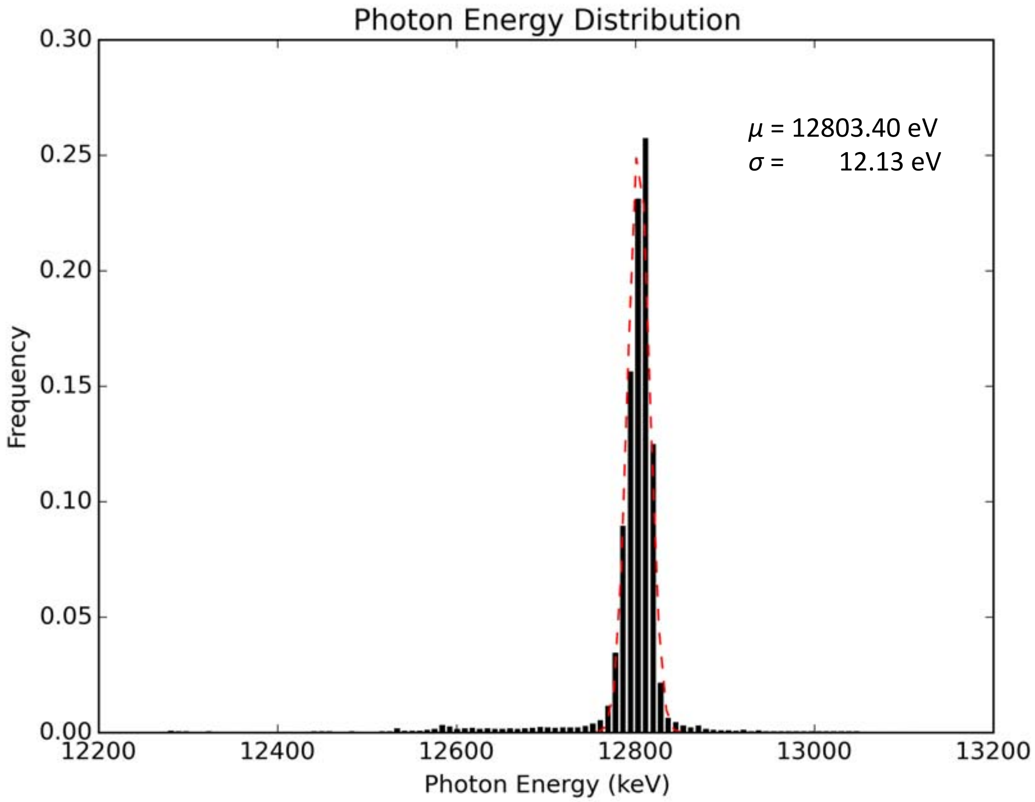

Supplementary Figure 4: Photon Energy Distribution. The photon energy distribution for X-rays delivered to the CXI hutch while data were being recorded.

	Number of frames Front / Back	Number of hits Front / Back	Number of indexed Front / Back
Transmission 100%	2,520,580 / 1,174,470	324,803 / 369,691	125,755 / 88,289
Transmission 50%	1,026,168 / 1,109,319	239,442 / 377,793	115,993 / 158,865
Transmission 10%	1,041,249 / 730,055	111,444 / 144,620	43,188 / 27,104
Total	7,601,841	1,567,793	559,194

Supplementary Table | Hit finding / indexing summary

Supplementary Note 1

For the large data sets acquired in SFX, the metric R_{split} is used to test the internal consistency of the data[1] by calculating the R-factor,

$$R_{\mathrm{split}} = \frac{1}{\sqrt{2}} \frac{\sum_{hkl} \left[I_{hkl}^{\mathrm{subset\_1}} - I_{hkl}^{\mathrm{subset\_2}} \right]}{\sum_{hkl} \left[\frac{I_{hkl}^{\mathrm{subset\_1}} + I_{hkl}^{\mathrm{subset\_2}}}{2} \right]}$$

between the data sets. For the final data set used to phase the Se-B SA structure with resolution cutoff at 1.9 Å, the R_{split} was calculated to be 4.8%, showing good internal consistency of the data.

References

1. White TA, *et al.* CrystFEL: a software suite for snapshot serial crystallography. *Journal of Applied Crystallography* **45**, 335-341 (2012).
